# Hairy Cell Leukemia: Clinical Characteristics and Outcomes from a Single Center in the Middle East and North Africa

**DOI:** 10.2147/JBM.S595093

**Published:** 2026-05-20

**Authors:** Shehab Mohamed, Mohamad Wajeh Dulli, Abdulrahman Al-Mashdali, Rola Ghasoub, Mohammed Abdulgayoom, Honar Cherif, Susanna Akiki, Anil Elahie, Deena Mudawi, Hesham Elsabah, Mohamed Yassin, Noor Salah Moustafa, Leen Haj Saleh, Priyadarshini Asmita Vatsayana, Samah Kohla, Firyal Ibrahim, Dina Soliman

**Affiliations:** 1 Department of Hematology and Bone Marrow Transplant, National Center for Cancer Care and Research, Hamad Medical Corporation, Doha, Qatar; 2 Department of Internal Medicine, Hamad General Hospital, Hamad Medical Corporation, Doha, Qatar; 3 Department of Pharmacy, National Center for Cancer Care and Research, Hamad Medical Corporation, Doha, Qatar; 4 Department of Laboratory Medicine and Pathology, Haemato-Oncology Diagnostic Genomic Division, Hamad Medical Corporation, Doha, Qatar; 5 College of Medicine, Qatar University, Doha, Qatar; 6 Corporate Quality Department, National Center for Cancer Care and Research, Hamad Medical Corporation, Doha, Qatar; 7 Department of Laboratory Medicine and Pathology, Hematopathology Section, Hamad Medical Corporation, Doha, Qatar; 8 College of Medicine, Al-Azhar University, Cairo, Egypt; 9 Weill Cornell Medicine - Qatar, Weill Cornell Medicine of Cornell University, Doha, Qatar; 10 Department of Clinical Pathology, National Cancer Institute, Cairo University, Cairo, Egypt

**Keywords:** hairy cell leukemia, BRAF V600E, purine analogues, cladribine, immunophenotyping, cytogenetics, Middle East, rituximab

## Abstract

**Purpose:**

Hairy Cell Leukemia (HCL) is a rare, chronic B-cell lymphoproliferative neoplasm characterized by the accumulation of abnormal lymphocytes in the bone marrow and spleen. Although outcomes are generally favorable with current therapies, data from diverse geographic and ethnic populations remain limited. This study highlights the disease clinicopathologic characteristics, treatment, and outcome within one center in the Middle East.

**Methods:**

A retrospective analysis was conducted on patients diagnosed with HCL at a tertiary care center, meeting the 5th edition WHO and ICC 2022 diagnostic criteria. Clinical, laboratory, and pathological data were extracted from institutional records.

**Results:**

Twenty-two patients were identified. The cohort represented diverse nationalities across Asia, Africa, Europe, and North America. Most cases were detected incidentally, while others presented with constitutional symptoms, abdominal pain, or febrile neutropenia. All but one case were classic HCL. Most expressed Pan B-cell markers, though a few showed aberrant loss of CD19, PAX-5, or CD103. The BRAF V600E mutation was detected in 82% of the tested cases. Treatment regimens included Cladribine alone or with Rituximab, yielding high response rates and minimal toxicity. Five-year overall survival was 100%.

**Conclusion:**

In this multiethnic Middle Eastern and North African cohort, HCL predominantly affected middle-aged men and was often incidentally detected. Cladribine-based therapy achieved durable remissions with excellent survival, highlighting consistent efficacy across diverse populations.

## Introduction

Hairy Cell Leukemia (HCL) is a rare, indolent B-cell lymphoproliferative disorder first characterized in the 1950s and later well-defined through clinical and pathological studies. It accounts for approximately 2% of all leukemias, with an estimated incidence of 0.3 cases per 100,000 individuals per year, predominantly affecting middle-aged men. The disease is characterized morphologically by lymphocytes with cytoplasmic projections (the eponymous “hairy” appearance), and clinically by pancytopenia, splenomegaly, and recurrent infections.^1^

In the most recent WHO edition, HCL was included under the umbrella of splenic B-cell lymphoma/leukaemia, and the Hairy cell leukaemia variant was omitted and replaced by Splenic B-cell leukaemia/lymphoma with prominent nucleoli (SBCLPN). On the other hand, the International Consensus Classification (ICC) retained both HCL and HCL-variant in its latest classification. To support flow cytometry-based diagnosis and differentiation from HCL-like disorders, an immunological HCL score has been established, assigning one point for each of four key markers (CD11c, CD25, CD103, and CD123) when expressed. A score of 3 or 4 is observed in approximately 98% of classic HCL cases, while HCL-V and splenic diffuse red pulp lymphoma (SDRPL) typically score 0 or 1, reflecting their lack of CD25 and CD123 expression.^2^

Classic HCL is strongly associated with the BRAF V600E mutation, present in more than 95% of cases, which plays a pivotal role in its pathogenesis and serves as both a diagnostic and potential therapeutic target. Hairy cell leukemia variant (HCL-V), retained in the ICC 2022 classification as a distinct entity, typically lacks the BRAF V600E mutation, expresses CD11c but not CD25, and follows a more aggressive clinical course.^3^,^4^ Diagnostic confirmation requires a combination of peripheral blood morphology, bone marrow biopsy, flow cytometry (highlighting markers such as CD11c, CD25, CD103, and annexin A1), and molecular testing. Cytogenetic abnormalities are rare but may be observed, including occasional CCND1/IGH fusions and TP53 mutations, which may have prognostic implications.^5^

Recent updates, including consensus guidelines from the international HCL community, underscore the need for precise diagnosis, risk stratification based on clinical and molecular characteristics, and a personalized treatment approach. However, data from underrepresented regions and real-world clinical settings remain scarce, particularly in diverse ethnic populations.^6^

In this study, we describe our single-centre experience from Qatar, detailing the clinicopathologic features, treatment outcomes, and molecular profiles of patients diagnosed with HCL. A preliminary abstract of this work has been previously presented.^7^ Qatar has a population of approximately 2.7 million and is characterized by a highly diverse population with a large expatriate community. In our practice, however, the majority of patients are of Asian background, with a male predominance. Our institution is the only tertiary cancer center in the country and serves as the national referral center for hematologic malignancies. By presenting data from a multiethnic Middle Eastern cohort, this analysis aims to enhance the global understanding of HCL and shed light on both typical and atypical disease presentations observed in this unique population.

## Methods

### Study Design and Setting

We conducted a retrospective, single-center cohort study at a tertiary cancer center in Qatar (The National Centre for Cancer Care and Research, NCCCR). All consecutive patients with a pathologically confirmed diagnosis of classic hairy cell leukemia (HCL) who received care at our institution between 2013 and 2025 were eligible. The study followed STROBE guidance for observational studies. Patients with suspected hairy cell leukemia, based on clinical findings such as splenomegaly or laboratory abnormalities including peripheral blood smear findings, are referred to our center for definitive diagnosis and management. Diagnostic evaluation typically includes flow cytometry, bone marrow examination, and molecular testing when indicated. In patients requiring treatment, cladribine is our main first-line therapy when clinically appropriate.

### Eligibility Criteria

#### Inclusion

Adults (≥18 years) with a new or established diagnosis of HCL (either classic or variant based on the latest ICC criteria) according to WHO/ICC criteria, supported by morphology and immunophenotype (typical “hairy” lymphocytes and expression of CD19, CD20, CD22 with co-expression of CD11c, CD25, CD103 and/or CD123) and, when available, molecular confirmation of *BRAF V600E.*

#### Exclusion

Other splenic B-cell lymphomas mimicking HCL, insufficient records to confirm diagnosis, or inadequate follow-up data.

### Data Sources and Variables

Clinical, laboratory, and pathologic data were retrospectively obtained from electronic medical records and institutional pathology databases. Collected variables included demographics (age, sex, nationality), presenting features (B-symptoms, infections, hepatosplenomegaly), baseline laboratory indices (hemoglobin, platelet, leukocyte, and neutrophil counts, LDH), bone marrow morphology and flow cytometry findings, and available cytogenetic or molecular results. Treatment data included the first-line regimen, the number of cycles, and treatment-related adverse events. Outcomes included hematologic recovery, radiologic response (when available), infectious complications, and dates of relapse, progression, last follow-up, or death.

### Outcomes and Definitions

#### Primary Outcome

Progression-free survival (PFS), defined as the time from date of diagnosis to first documented relapse/progression or death from any cause, whichever occurred first. Patients without an event were censored at the last clinical contact.

#### Secondary Outcomes

Overall survival (OS) (diagnosis→death from any cause; censored at last contact), overall/complete response rates after initial therapy (per institutional practice aligned with consensus criteria: normalization of counts without support and absence of morphologic disease in blood and marrow.

#### Relapse/Progression

Reappearance of cytopenias and/or morphologic/flow/PCR evidence of HCL requiring treatment, after a documented complete/partial response.

### Statistical Analysis

Continuous variables are reported as mean ± SD or median (IQR); categorical variables as counts (%). Denominators vary between analyses, reflecting only patients with available data for each test.

### Data Management and Quality Control

Two investigators independently abstracted key dates (diagnosis, relapse/progression, last follow-up, death) and cross-checked pathology and treatment data; discrepancies were resolved by consensus with a third reviewer. Dates were stored in ISO-8601 format; derived intervals (days, months) were computed programmatically with audit trails.

## Results

A total of 22 patients with hairy cell leukemia (HCL) were included. The median age at diagnosis was 51 years (range, 37–89), and most patients were male (19/22, 86%). Nationalities were predominantly South Asian and Middle Eastern, with Indians (n=5) and Pakistanis (n=3) forming the largest groups, followed by Lebanese and Egyptian patients (n=2 each); the remaining patients were from various European, North African, and North American backgrounds (Table 1).

**Table 1:** Baseline Demographics, Clinical Characteristics, Laboratory Findings, and Treatment Regimens

**Demographics**	**No:**	**%**
Male	19	86%
Female	3	14%
Asian	9	41%
Middle Eastern/Arab	10	45%
European	2	9%
North American	1	5%
Manifestations *		
Asymptomatic	9	43%
Fatigue, Dizziness, Weight loss	3	14%
Febrile neutropenia	1	5%
Neutropenic sepsis	1	5%
Pathological fracture, Mediastinal mass	1	5%
Shortness of breath, Abdominal distention	1	5%
Vertebrae fractures	1	5%
COVID-19, ARDS	1	5%
Cryptococcal meningitis	1	5%
Abdominal pain	1	5%
Night sweats	1	5%
Splenomegaly (n=21)	14/21	67%
Hepatomegaly (n=21)	4/21	19%
**Blood Workup**		
**Blood Test**	**Average**	**Standard Deviation**
WBC (×10^9^/L)	5.6	7.95
Hb (g/dL)	12.04	3.05
ANC (×10^9^/L)	1.29	0.68
ALC (×10^9^/L)	2.01	1.15
AMC (×10^9^/L)	1.59	2.3
Plt (×10^9^/L)	88.3	39.6
LDH (U/L)	214.45	157.4
**Treatment Protocol, Side Effects**		
**Treatment**	**Number (%)**	
	**No:**	**%**
Cladribine + Rituximab	9	56%
Cladribine	6	38%
Pentostatin	1	6%

**Notes**: * Patients may present with more than one clinical manifestation.

**Abbreviations**: WBC, white blood cells; Hb, hemoglobin; ANC, absolute neutrophil count; ALC, absolute lymphocyte count; AMC, absolute monocyte count; Plt, platelets; LDH, lactate dehydrogenase.

Of the 22 patients included, 21 were classified as classic HCL and 1 as HCL-variant (HCL-V) based on immunophenotypic and molecular features.

Clinical presentation was heterogeneous. Nine patients (41%) were asymptomatic at diagnosis, most commonly identified during evaluation of cytopenias or incidental splenomegaly. Twelve patients (55%) were symptomatic, with features including abdominal pain, fatigue, weight loss, night sweats, dyspnea, pathological or vertebral compression fractures, and infection-related syndromes such as febrile neutropenia, neutropenic sepsis, cryptococcal meningitis, and COVID-19–related acute respiratory distress syndrome; symptoms were not documented for one patient. Radiologically, splenomegaly was present in 14 of 21 patients (67%) with available ultrasound data, with splenic length typically 13–23 cm; seven had no splenomegaly and one had missing data. Hepatomegaly was reported in 4 of 21 patients (19%), while 17 had no hepatomegaly and one had missing data.

Baseline blood counts showed a median white blood cell count of 3.7 ×10^9^/L (range, 0.3–38.6), median hemoglobin of 12.6 g/dL (range, 5.0–17.6), and median platelet count of 80 ×10^9^/L (range, 49–214). Anemia (Hb <12 g/dL) was present in 8/21 patients (38%), and Hb <10 g/dL in 4/21 (19%). Neutropenia (ANC <1.0 ×10^9^/L) occurred in 7/21 (33%), including 2 with severe neutropenia (ANC <0.5 ×10^9^/L). Thrombocytopenia was the most prominent cytopenia: 19/21 (90%) had platelets <150 ×10^9^/L and 15/21 (71%) had platelets <100 ×10^9^/L.

Bone marrow reports were available for 19 patients and showed the typical dense hairy-cell infiltration. Based on descriptive estimates, the median marrow involvement was approximately 80% (range, 30–100%), with most patients having 65–95% infiltration by abnormal hairy cells. Annexin A1 (ANXA1) immunohistochemistry was performed in a subset of cases where available in the pathology records and was positive, consistent with classic HCL. VE1 immunohistochemistry (a surrogate marker for BRAF V600E protein expression) was not systematically performed as a routine diagnostic test at our institution during the study period; in the five patients who did not undergo molecular BRAF V600E testing, diagnosis was established on the basis of morphology, immunophenotype (including expression of CD11c, CD25, CD103, and CD123 where applicable), and bone marrow histology, in keeping with WHO/ICC diagnostic criteria.

**Figure 1:**
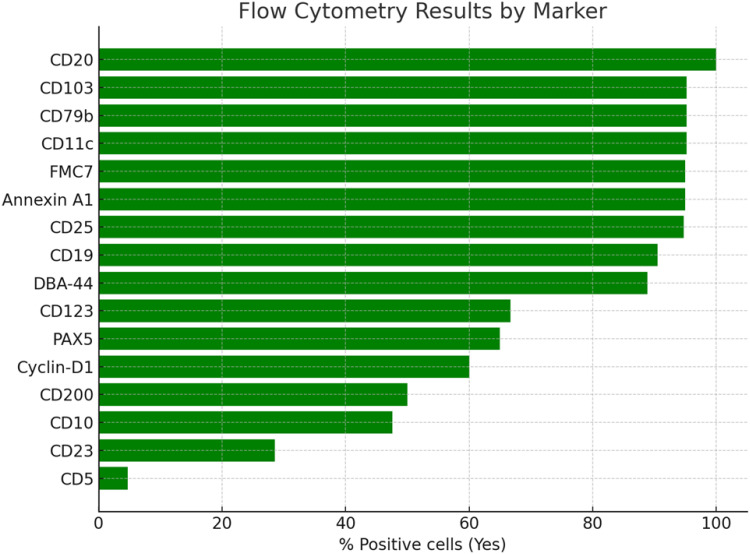
Flow cytometry marker positivity among patients with hairy cell leukemia.

**Figure 2:**
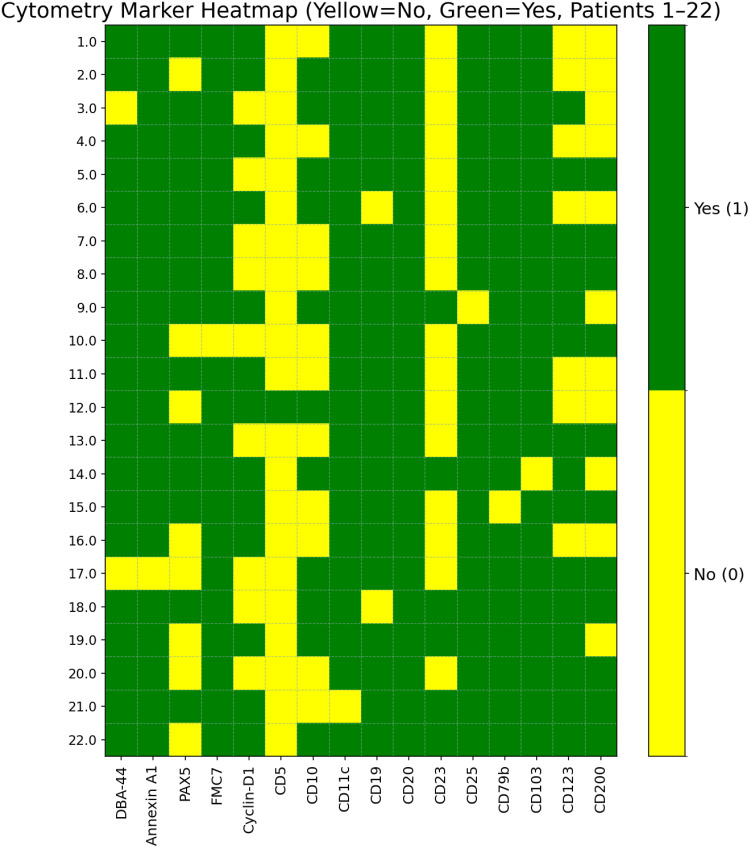
Heatmap of flow cytometry marker expression across individual cases.

Flow cytometry demonstrated strong expression of classic HCL markers, including CD19, CD20, CD22, CD103, CD25, CD11c, and CD123. CD11c was positive in 20 patients (95%). CD25 and CD103 were each negative in one patient, and CD123 was negative in 7 patients (31%). Marker positivity across the cohort is summarized in Figure 1, and case-level marker expression is shown in Figure 2.

Cytogenetic analysis showed a normal karyotype in 17 patients (77%). One patient had a t(11;14)(q13;q32) CCND1/IGH rearrangement, an aberration more typical of mantle cell lymphoma. The patient with HCL-variant (HCL-V) demonstrated hypertetraploidy and TP53 deletion, together with negative CD25 and CD200 expression and downregulation of CD123. Molecular testing for BRAF V600E was performed in 17 patients and was positive in 14 (82%) and negative in 3 (18%), consistent with the expected predominance of BRAF-mutated classic HCL. BRAF V600E was not routinely re-tested at response assessment in this retrospective cohort. Complete remission (CR) was defined per institutional practice, aligned with published consensus criteria: normalization of peripheral blood counts (hemoglobin ≥11 g/dL, neutrophils ≥1.5×10^9^/L, platelets ≥100×10^9^/L) without transfusion or growth factor support, and absence of morphologic hairy cell infiltration on bone marrow biopsy.^6^ Bone marrow re-biopsy was performed to confirm CR in patients where clinical assessment and blood count normalization warranted it, though MRD testing by flow cytometry or PCR was not systematically applied.^8^

First-line systemic therapy data were available for most patients. Sixteen of 22 (73%) received a purine analogue–based regimen: cladribine alone in 6 (27%), cladribine plus rituximab in 9 (41%), and pentostatin in 1 (5%). All treated patients achieved complete remission.

Treatment-related toxicity was mainly infectious. Febrile neutropenia occurred in 7 patients, and one additional patient experienced a complex toxicity profile including rituximab infusion reaction, non-tuberculous mycobacterial infection, confusion, and cytomegalovirus reactivation, resulting in 8/22 patients (36%) with significant acute treatment-related adverse events. No long-term toxicities were systematically captured.

Progression-free survival (PFS) was evaluable in 17 patients, with an estimated 5-year PFS of 90%. At a median follow-up of 27 months (range, 4–96 months), 15/17 patients (88%) remained relapse-free, while 2 (12%) had relapsed. All 22 patients were alive at the last follow-up.

## Discussion

Our single-center experience at the NCCCR - Qatar’s only tertiary cancer and hematology referral center, adds to the limited data on hairy cell leukemia (HCL) from the Middle East and Gulf region and broadly aligns with the established epidemiologic profile described in Western and non-Western cohorts. Reported incidence rates of 0.3–0.6 per 100,000, with a marked male predominance and median age in the mid-50s, are consistent across large series from Europe and North America, as well as smaller cohorts from Italy and Iraq.^1^,^9^ Our median age is in the early 50s, and the male-to-female ratio mirrors these findings.^1^,^9^ Notably, although our sample size is small, the population is highly diverse, including patients from Asia, Africa, Europe, North America, and multiple Arab countries, providing insight into HCL in a multiethnic Gulf setting. This series is, to our knowledge, the first from Qatar and only the third reported from the wider Middle East.^1^,^10^

The clinical presentation in our cohort was typical in many respects. As in prior studies, patients frequently presented with cytopenia-related symptoms, B symptoms, and splenomegaly, which has been reported in more than 80% of cases.^1^,^6^,^11^ In our series, around two-thirds had splenomegaly, and most showed significant cytopenias, again in keeping with published data.^1^,^11^ A substantial proportion, however, were asymptomatic and diagnosed incidentally, underlining the importance of routine blood counts and imaging in case detection. Elderly patients represented a minority but included an individual diagnosed at 89 years, illustrating that HCL can be encountered even in very advanced age, where treatment decisions and infection risk under purine analogue therapy require particular caution.^12^,^13^

A strength of our cohort is the documentation of atypical and rare presentations that can complicate diagnosis. Bone involvement with pathological fractures, although uncommon, has been described in case reports and small series,^11^,^14^ and one such case from our center has previously been published.^15^ Similarly, mediastinal lymphadenopathy - rare in HCL - can mimic other lymphoid malignancies;^16^ in our series, one patient presented with both a pathological fracture and a mediastinal mass. Opportunistic infections, including atypical mycobacterial disease and cryptococcal infection, were also observed, consistent with prior reports linking HCL and its treatment to unusual infections.^17–19^ These observations emphasize the need for a high index of suspicion for HCL in patients with unexplained cytopenias, bone lesions, or opportunistic infections, particularly in immunocompromised or heavily pretreated individuals.

Our bone marrow and immunophenotypic findings largely corroborate the classic diagnostic profile of HCL. The high marrow infiltration burden in most cases is consistent with prior studies reporting median involvement around 80–85%.^1^,^6^,^20^ Immunophenotypically, our cases showed robust expression of B-cell markers and typical HCL antigens such as CD103, CD11c, CD25, and CD123, alongside expression of CD200 and Cyclin D1 in a subset.^2^,^21^ However, we also observed immunophenotypic variability, including expression of CD5, CD10, and CD23 in small proportions of patients, and occasional loss of markers such as CD25 or CD103. Of particular note, CD5 positivity - an antigen classically associated with CLL and mantle cell lymphoma - was observed in a minority of cases and underscores the importance of a comprehensive immunophenotypic panel.^21^,^22^ CD123 negativity, seen in nearly a third of our cohort, may raise the differential of HCL-V and should prompt careful morphologic and molecular correlation, as CD123 expression is typically retained in classic HCL but absent in HCL-V.^23^ Such atypical immunophenotypes have been increasingly recognized and may lead to diagnostic confusion with other B-cell neoplasms, highlighting the need to interpret flow cytometry in the context of morphology, clinical features, and molecular data.^21^,^24–28^ Our findings underscore that immunophenotypic heterogeneity is not rare in real-world practice and should be anticipated, particularly in referral centers.

Cytogenetic and molecular data in our series further support the concept that while most HCL cases have normal karyotypes, a subset harbor additional abnormalities. Prior work has shown that cytogenetic abnormalities occur in about 10% of newly diagnosed patients and do not necessarily impact overall survival.^28^ In our cohort, most patients had normal karyotypes, but we observed TP53 deletions and CCND1/IGH rearrangements in a few cases. TP53 lesions have been associated with more aggressive behavior and suboptimal responses in some series,^4^,^29^ whereas Cyclin D1 expression and t(11;14) are classically associated with mantle cell lymphoma rather than HCL. The coexistence of BRAF V600E, CCND1/IGH rearrangement, and TP53 deletion in one of our patients supports the interpretation of a composite lymphoma involving HCL and mantle cell lymphoma, as previously described in rare reports.^30^ These observations highlight the importance of integrating cytogenetics, FISH, and molecular data in diagnostically challenging or atypical cases.

Consistent with prior literature, the BRAF V600E mutation was detected in the majority of our tested patients, with a positivity rate comparable to that reported in other cohorts, though somewhat lower than the >95% frequency described in some series.^2^,^4^,^31^ The three BRAF-negative cases in our cohort may reflect genuine biological heterogeneity or, alternatively, technical limitations related to low disease burden at the time of testing. The identification of BRAF V600E in both HCL and papillary thyroid carcinoma in one patient is particularly notable and echoes reports of BRAF-driven oncogenesis across multiple malignancies.^32^,^33^

From a therapeutic standpoint, our experience supports purine analogue–based therapy, particularly cladribine with or without rituximab, as a highly effective first-line treatment, in line with previous Phase II data reporting high complete remission rates and durable responses.^8^,^34^,^35^ All treated patients in our cohort achieved complete remission, and relapse rates were low over the available follow-up.

The addition of rituximab to cladribine (CDA+R) has been shown in prospective studies to improve minimal residual disease (MRD) negativity rates and prolong remission duration compared with cladribine alone, although the survival benefit remains under study.^36^,^37^ In our cohort, the majority of treated patients received CDA+R, which may have contributed to the low relapse rate observed. In the event of relapse, treatment options include re-treatment with purine analogs, BRAF-targeted therapy with vemurafenib (for BRAF V600E-positive cases), and the anti-CD22 immunotoxin moxetumomab pasudotox.^38^,^39^

However, infectious complications - including febrile neutropenia, opportunistic infections, and rituximab infusion reactions - were relatively frequent, echoing previous reports and underscoring the need for careful infectious risk assessment and prophylaxis where appropriate.^17–19^ Despite these toxicities, most patients maintained good performance status post-therapy.

IGHV mutational status was not systematically evaluated in this cohort; future studies should include this analysis, as emerging data suggest that IGHV usage patterns in HCL may differ from those in other B-cell neoplasms and could have implications for pathogenesis and response to therapy.^29^

This study has important limitations. The small sample size (22 patients), incomplete molecular testing in all patients, heterogeneous follow-up, and absence of routine MRD monitoring restrict the precision of our estimates and the generalizability of our findings. Nevertheless, this is the first detailed HCL report from Qatar and one of the few from the Middle East,^1^,^10^ contributing region-specific data from a highly diverse, multiethnic cohort. The systematic documentation of atypical presentations, rare cytogenetic findings, and real-world toxicity profiles adds clinically relevant information for practitioners in similar settings.

## Conclusion

Our single-center experience confirms that HCL in a Middle Eastern, multiethnic population broadly mirrors the clinical, morphologic, and molecular features described in Western series, while also highlighting atypical presentations and complex cytogenetic constellations. Cladribine-based regimens, with or without rituximab, achieved excellent responses and favorable outcomes, but infectious complications remain a key concern. Future multicenter, prospective studies incorporating standardized MRD assessment and comprehensive molecular profiling are needed to refine risk stratification and optimize management of both typical and atypical HCL in this region. HCL thus remains a rare but highly treatable malignancy when promptly recognized and appropriately managed.
